# Haplotype analysis identifies functional elements in monoclonal gammopathy of unknown significance

**DOI:** 10.1038/s41408-024-01121-8

**Published:** 2024-08-20

**Authors:** Hauke Thomsen, Subhayan Chattopadhyay, Niels Weinhold, Pavel Vodicka, Ludmila Vodickova, Per Hoffmann, Markus M. Nöthen, Karl-Heinz Jöckel, Börge Schmidt, Roman Hajek, Göran Hallmans, Ulrika Pettersson-Kymmer, Florentin Späth, Hartmut Goldschmidt, Kari Hemminki, Asta Försti

**Affiliations:** 1 MSB Medical School Berlin, Hochschule für Gesundheit und Medizin, Berlin, Germany; 2 Division of Clinical Genetics, Department of Laboratory Medicine, Lund University, Lund, Sweden; 3 Department of Internal Medicine V, University of Heidelberg, Heidelberg, Germany; 4 Institute of Experimental Medicine, Academy of Sciences of the Czech Republic, Prague, Czech Republic; 5 Institute of Biology and Medical Genetics, 1st Medical Faculty, Charles University, Prague, Czech Republic; 6 Faculty of Medicine and Biomedical Center in Pilsen, Charles University in Prague, Pilsen, Czech Republic; 7 Institute of Human Genetics, University of Bonn, Bonn, Germany; 8 Department of Biomedicine, University of Basel, Basel, Switzerland; 9 Institute for Medical Informatics, Biometry and Epidemiology, University Hospital Essen, University of Duisburg-Essen, Essen, Germany; 10 Department of Hematooncology, University Hospital Ostrava and Faculty of Medicine, University of Ostrava, Ostrava, Czech Republic; 11 Department of Public Health and Clinical Medicine, Umea University, Umea, Sweden; 12 Clinical Pharmacology, Department of Pharmacology and Clinical Neuroscience, Umea University, Umea, Sweden; 13 Department of Diagnostics and Intervention, Cancer Center, Hematology, Umeå University, Umeå, Sweden; 14 National Centre of Tumor Diseases, Heidelberg, Germany; 15 Department of Cancer Epidemiology, German Cancer Research Center, Heidelberg, Germany; 16 Hopp Children’s Cancer Center (KiTZ), Heidelberg, Germany; 17 Division of Pediatric Neurooncology, German Cancer Research Center (DKFZ), German Cancer Consortium (DKTK), Heidelberg, Germany

**Keywords:** Cancer genetics, Cancer genetics

## Abstract

Genome-wide association studies (GWASs) based on common single nucleotide polymorphisms (SNPs) have identified several loci associated with the risk of monoclonal gammopathy of unknown significance (MGUS), a precursor condition for multiple myeloma (MM). We hypothesized that analyzing haplotypes might be more useful than analyzing individual SNPs, as it could identify functional chromosomal units that collectively contribute to MGUS risk. To test this hypothesis, we used data from our previous GWAS on 992 MGUS cases and 2910 controls from three European populations. We identified 23 haplotypes that were associated with the risk of MGUS at the genome-wide significance level (*p* < 5 × 10^−8^) and showed consistent results among all three populations. In 10 genomic regions, strong promoter, enhancer and regulatory element-related histone marks and their connections to target genes as well as genome segmentation data supported the importance of these regions in MGUS susceptibility. Several associated haplotypes affected pathways important for MM cell survival such as ubiquitin-proteasome system (*RNF186*, *OTUD3*), PI3K/AKT/mTOR (*HINT3*), innate immunity (*SEC14L1*, *ZBP1*), cell death regulation (*BID*) and NOTCH signaling (*RBPJ*). These pathways are important current therapeutic targets for MM, which may highlight the advantage of the haplotype approach homing to functional units.

## Introduction

Germline disease genetics has historically applied linkage studies between family members to find susceptibility genes. With time families have become smaller which has reduced the statistical power of family-based linkage studies [1]. However, with increasing understanding of the human genome organization, genetic variants, including single nucleotide polymorphisms (SNPs), have been identified at specific locations throughout the genome. SNPs are inherited together as haplotypes containing a linked sets of alleles and allele-specific biological functions, including genes and their cis-regulatory elements [2]. In the study on global reference for human genetic variation 3.53 million SNPs were used for the European population to define haplotypes [3]. One goal for the development of dense linkage map with well-defined haplotypes over the human genome has been the possibility to map susceptibility genes assuming that some marker SNPs would be in high linkage with the functional variants, even if the SNPs lacked independent functions [4]. This has been the driving idea behind the genome-wide association studies (GWASs) for which the SNP coverage increased hugely in a decade. The result has been that the current GWASs target increasing numbers of haplotypes and may identify functional variants in even rare haplotypes [5].

The importance of haplotypes has been well known in some cancers, particularly in lymphoma, where immune related haplotypes at the HLA locus are associated with disease risk or protection [6]. Other cancer-related applications of haplotypes include dating the origins of mutations based on conserved haplotypes [7]. However, direct use of haplotypes has not been popular in human germline genetics, in marked contrast to its role in animal breeding [8, 9]. Nevertheless haplotype-based genetic mapping has been used in some recent Swedish cancer studies where novel candidate genes have been detected [10, 11].

In the present study we apply a haplotype-based gene mapping approach to monoclonal gammopathy of unknown significance (MGUS) which is a precursor condition for multiple myeloma (MM) and other plasma cell malignancies such as Waldenström macroglobulinemia and immunoglobulin light chain (AL) amyloidosis. The MGUS populations included Swedish, German and Czech individuals and local controls who have been genotyped in previous GWASs [12–15]. For haplotype analysis diverse populations may be a disadvantage as the haplotype structure may show subtle differences. However, the advantage is the increase in sample size and the possibility to internally replicate the findings in three populations.

## Methods

### Populations and original GWASs

We included three independent GWAS data sets of MGUS patients and controls from Germany, the Czech Republic and Sweden, including a total of 992 MGUS cases and 2910 controls, as described elsewhere [15]. Collection of patient samples and associated clinical information was undertaken with informed consent in accordance with the tenets of the Declaration of Helsinki. The study was approved by the Ethical Committees of the universities of Heidelberg, Ostrava and Umea. The diagnosis of MGUS was based on the internationally accepted criteria of a monoclonal protein concentration <30 g/L, <10% monoclonal plasma cells in the bone marrow, normal plasma calcium and normal renal function, no bone destruction and no anemia.

Details of the individual GWASs and imputation have been described earlier [15]. Shortly, the German MGUS set was genotyped with Illumina Human OmniExpress-12 v1.1 arrays and the control set with Illumina Human OmniExpress-12 v1.0 arrays. The Czech individuals were genotyped using Illumina HumanOmniExpressExome8v1.3 arrays. The Swedish samples were genotyped with Illumina Human Omni1-Quad BeadChips or OmniExpress-12 v1.0 arrays. General quality control assessment of genotyping was performed as previously described by Broderick et al. [16] and Chubb et al. [17]. SNPs with a minor allele frequency (MAF) < 5% were excluded due to statistical uncertainty at lower allele frequencies using the current population sizes. After imputation using data from the combined UK10K - 1000 Genomes Project (Phase 3, Oct 2014) with IMPUTE2 v2.3.2 [18], the genotyped and imputed SNP sets consisted of 4 015 889 SNPs for the Czech population, 5,305,950 SNPs for the German population, and 4,455,885 SNPs for the Swedish population. All genomic positions are given in NCBI Build 37/UCSC hg19 coordinates.

### Construction of haplotype blocks

Haplotype blocks were created for each population separately based on 13,777,724 SNPs for a total of 3902 subjects. For construction of the haplotype blocks, two steps were followed: 1) phasing of chromosomes through SHAPEIT.v2 [19] and 2) construction of haplotype blocks through the “Ghap” package in R [20].

For phasing, the genotype data was segregated based on chromosomes with creation of individual chromosome binary ped and map files by using the PLINK program [21]. Chromosome-wide genotypes were then phased again using SHAPEIT.v2.12 with 200 states and window size of 0.5 Mb. SHAPEIT has been acknowledged to be most efficient for estimating haplotypes from the genotypes and for creating the consecutive haps and sample file which were used to generate haplotype blocks through GHap.

Haplotype blocks were constructed based on linkage disequilibrium (LD), Length (Len), and the number of SNPs (nsnp) and corresponding phase, sample, and marker files for each chromosome were created. Genomic positions in haplotype blocks were based on the distance from the first SNP. After phasing, haplotype blocks were constructed using the GHap function, which generates HapBlocks based on sliding windows. Windows and the step size can be specified in markers. For each window, block coordinates are generated. Based on the fact that the average length of linkage disequilibrium (LD) blocks in human populations is about 20 kb [22, 23] the window size was defined to be 15 SNPs in order to cover a similar span. The sliding block along the genome was defined to be 2 SNPs during the call haplotypes. For each window, block coordinates were generated as the mean position of the start and end position of the corresponding haplotype window.

Overall, a total of 99,518,698 haplotype blocks were created using the above-mentioned methods based on LD, the length and the number of SNPs. In a consecutive step, we used GHap package in R to derive haplotype “alleles” from these haplotypes’ blocks. Haplotype “alleles” were then exported in the PLINK ped file format, where haplotype allele counts 0, 1, and 2 are recoded as NN, NH, and HH genotypes (H = haplotype allele and N = NULL = all other alleles), as if haplotypes were bi-allelic markers. A regular ped file was obtained with PLINK and the haplotypes presenting a MAF < 0.01 were filtered out. These data sets were further used to perform a haplotype-based association study.

### GWAS of the haplotypes

Haplotype alleles determined by GHap were used for association studies conducted by PLINK using logistic regression models with the covariate sex. The association analysis was first performed for each single population based on the haplotypes derived in the steps above.

### Meta-analysis

Meta-analysis was performed using PLINK by pooling the beta values and standard errors for haplotypes from each single population data set. The meta-analysis was performed with a random effects inverse-variance weighted logistic model. Cochran’s Q-statistic was calculated, to test for heterogeneity, and the I2 statistic measured, to quantify the proportion of the total variation due to heterogeneity.

**Fig. 1:**
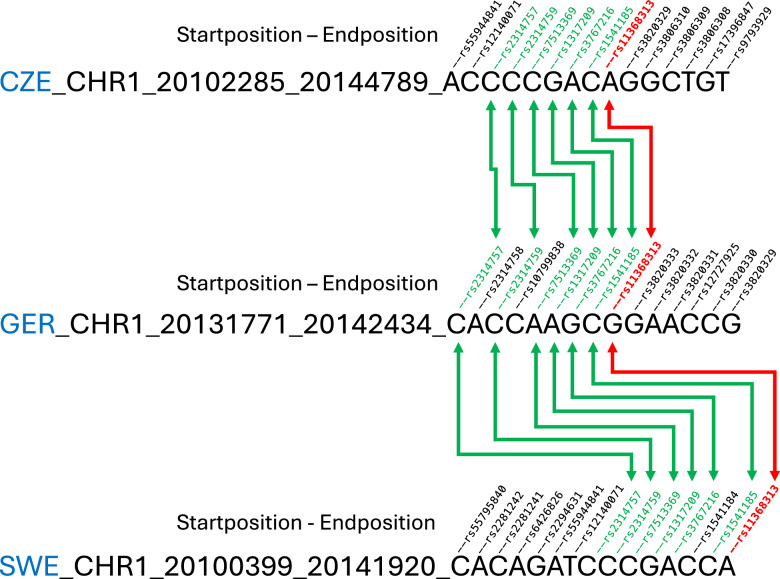
Meta-analysis of the haplotypes from the three populations, originating from the Czech Republic (CZE), Germany (GER) and Sweden (SWE), represented by the joint SNP rs11368313 (in red). Other SNPs common in all three populations are shown in green. For each population, the chromosome start and end position and the allele of the haplotype are shown.

While normally the meta-analysis of GWAS data from different populations refers to common SNPs in the different data sets, this approach does not work for haplotypes, since only in rare cases exactly identical haplotypes could be derived in all populations. As an alternative, we therefore used a common SNP in the corresponding haplotypes in the three populations as an anchor point for the analysis (from now on referred to as “a joint SNP”). For a haplotype in each population, all SNPs forming the haplotype were assigned and a meta-analysis was performed for the joint SNP to represent each haplotype (Fig. 1). However, the joint SNP is only used as a common anchor and it represents the P value, odds ratio and frequency of the haplotype. In case of a large number of common SNPs in a haplotype, the most significant one was assigned as the joint SNP. Similarly, if common SNPs along the chromosome were assigned to several overlapping haplotypes, the haplotype represented by a joint SNP with the highest significance was selected.

### Gene identification in significant haplotype blocks

Haplotypes that after meta-analysis of the three populations were associated with the risk of MGUS at the genome-wide significance level *p* < 5 × 10^−8^ and showed increased risk of MGUS in all three populations were investigated further using UCSC browser’s GRCh37/hg19 assembly (https://genome.ucsc.edu) [24, 25]. Overlapping haplotypes were defined by a joint SNP and the start and end positions of the haplotype regions were based on the lowest start and highest end positions, among the overlapping haplotypes. We included tracks of UCSC genes, Genome Segmentations and Histone modifications by ChIP-seq from ENCODE [26], CpG Islands, Transcription Factor ChIP-seq Clusters from ENCODE, Enhancers and promoters from GeneHancer (Double Elite) [27] and Interactions between GeneHancer regulatory elements and genes (Double Elite). For Histone modification tracks we included H3K27Ac as an enhancer marker, H3K4me3 as a promoter and transcribed region marker and H3K4Me1 as regulatory region marker from the GM12878 lymphoblastoid cell line from ENCODE. Further information about the associated GeneHancer promoters and enhancers were extracted via GeneCards (https://www.genecards.org). Our focus was on the Double Elite regulatory elements and associated genes, i.e. they were confirmed by several sources used by GeneHancer. We used the Roadmap Epigenomics project data on histone marks to evaluate the regions in different immune cell populations, including primary B-cells, monocytes, hematopoietic stem cells, neutrophils, T-cells and natural killer cells (https://epigenomegateway.wustl.edu/) [28]. We also evaluated the histone marks in other adult cells and tissues, including ovary, adipose nuclei, osteoblasts, lung, breast myoepithelial cells, adult liver, thymus, skeletal muscle, spleen and gastrointestinal tract. We checked the function of the genes located within the haplotype regions and genes interacting with the regulatory elements within the haplotype regions using GeneCards (https://www.genecards.org) and UniProt (https://www.uniprot.org). We used PubMed (https://pubmed.ncbi.nlm.nih.gov) to look for the relationship between the genes with cancer, especially with multiple myeloma or other hematological malignancies.

## Results

We used a joint SNP as an anchor point to create overlapping haplotypes for the three populations of our study (Fig. 1). We identified 23 haplotypes that were associated with the risk of MGUS at the genome-wide significance level *p* < 5 × 10^−8^ and showed increased risk of MGUS in all three populations (Table 1). One region represented by the SNP rs111335312 was located within the *ULK4* gene, in a known MM risk locus. Altogether 13 haplotype regions were overlapping with genes or regulatory elements interacting with the neighboring genes (Tables 1, 2). In these haplotype regions most of the regulatory elements were active in B-cells and/or immune cells; some of them were even recognized as super-enhancers. All regulatory elements had several target genes and most of them contained tens to a few hundred transcription factor binding sites.

**Table 1:** Haplotype regions associated with the risk of MGUS.

Joint SNP	CHR	BP	OR	95% CI	p-value	Associated genes	Gene name	Function	GeneHancer interactions yes/no
rs11368313	1	20141882	3.59	2.37–5.43	1.56 × 10^−9^	TMCO2	Transmembrane and coiled-coil domains 2	Unknown; integral component of membrane	Yes
						RNF186	Ring finger protein 186	Apoptosis; ubiquitination	
						OTUD3	OTU deubiquitinase 3	Deubiquitination	
rs111335312	3	41777743	1.69	1.41–2.03	1.13 × 10^−8^	ULK4	unc-51 like kinase 4	Serine/threonine kinase	No
rs111723274	3	151675513	1.63	1.38–1.92	8.25 × 10^−9^	no gene			No
rs10938733	4	8676148	3.48	2.22–5.44	4.68 × 10^−8^	no gene			No
rs10006825	4	26153727	2.22	1.68–2.92	1.37 × 10^−8^	RBPJ	Recombination signal binding protein for immunoglobulin kappa J region	Regulator of Notch signaling; chromatin remodeling; binds to immunoglobulin kappa-type J segment recombination signal sequence	Yes
rs10940110	5	66794814	2.22	1.74–2.83	1.18 × 10^−10^	no gene			No
rs10514061	5	75435368	1.45	1.27–1.65	4.19 × 10^−8^	SV2C	Synaptic vesicle glycoprotein 2C	Neurotransmitter transport	No
rs10658790	6	126275488	1.59	1.36–1.86	6.29 × 10^−9^	HINT3	Histidine triad nucleotide binding protein 3	Nucleotide hydrolase and transferase	Yes
						TRMT11	tRNA methyltransferase 11 homolog	tRNA methylation	
						NCOA7	Nuclear receptor coactivator 7	Positive regulation of transcription by RNA polymerase II; coactivation of different nuclear receptors	
rs10253191	7	67372454	2.47	1.78–3.41	4.76 × 10^−8^	no gene			No
rs112048508	10	30443336	1.72	1.42–2.08	2.81 × 10^−8^	no gene			No
rs10567579	10	134807240	1.71	1.41–2.06	2.62 × 10^−8^	LINC01166; LINC01168	Long intergenic non-protein coding RNA	lncRNA	Yes
rs10628082	11	134612753	1.78	1.47–2.15	2.39 × 10^−9^	LOC105369584	Unknown locus		Yes
rs10840622	12	122288202	1.74	1.44–2.10	6.40 × 10^−9^	SETD1B	SET domain containing 1B, histone lysine methyltransferase	Chromatin remodeling; regulation of the transcriptional programming of multipotent hematopoietic progenitor cells and lymphoid lineage specification during hematopoiesis	No
						HPD	4-hydroxyphenylpyruvate dioxygenase	Tyrosine catabolism	
rs111792251	13	40895225	2.06	1.63–2.62	2.07 × 10^−9^	no gene			No
rs111282782	16	84564132	2.18	1.65–2.88	3.87 × 10^−8^	no gene			No
rs111362005	17	831933	3.84	2.38–6.20	3.44 × 10^−8^	NXN	Nucleoredoxin	Redox-dependent regulator of the Wnt signaling pathway; cell growth and differentiation	Yes
rs1024819	17	55607271	2.98	2.08–4.28	3.02 × 10^−9^	MSI2	Musashi RNA binding protein 2	Transcription regulation of genes involved in development and cell cycle	Yes
rs10163481	17	75148741	1.83	1.55–2.15	6.37 × 10^−13^	SEC14L1	SEC14 like lipid binding 1	Signal transduction inhibitor; innate immunity	Yes
						SNHG20	Small nucleolar RNA host gene 20	lncRNA	
						SRSF2	Serine and arginine rich splicing factor 2	mRNA splicing	
rs10543240	18	42189616	1.69	1.41–2.01	5.06 × 10^−9^	no gene			No
rs10420324	19	54469051	4.40	2.68–7.22	4.87 × 10^−9^	MYADM	Myeloid associated differentiation marker	Negative regulation of heterotypic cell-cell adhesion, macromolecule metabolic process and protein kinase C signaling	Yes
						PRKCG	Protein kinase C gamma	Expressed solely in the brain and spinal cord neurons	
						CACNG7	Calcium voltage-gated channel auxiliary subunit gamma 7	Regulation of the activity of L-type calcium channels	
						CACNG8	Calcium voltage-gated channel auxiliary subunit gamma 8	Regulation of the activity of L-type calcium channels	
						ZNF765	Zinc finger protein 765	Transcription factor	
						ZNF761	Zinc finger protein 761	Transcription factor	
						ZNF813	Zinc finger protein 813	Transcription factor	
						CNOT	CCR4-NOT transcription complex subunit 3	RNA-mediated gene silencing	
rs1051904	20	4163302	1.76	1.46–2.12	3.22 x 10^−9^	SMOX	Spermine oxidase	Oxidation of spermine to spermidine; determinant of cellular sensitivity to the antitumor polyamine analogs	Yes
rs111797554	20	56168743	2.18	1.66–2.85	1.52 × 10^−8^	ZBP1	Z-DNA binding protein 1	Innate immunity; apoptosis; necrosis	Yes
rs1045588	22	18271078	3.12	2.11–4.63	1.46 × 10^−8^	BID	BH3 interacting domain death agonist	BCL-2 family of cell death regulators	Yes
						MICAL3	Microtubule associated monooxygenase, calponin and LIM domain containing 3	Cell cycle; cell division; exocytosis

**Table 2:** GeneHancer promoters and enhancers in the haplotype regions associated with the risk of MGUS, details extracted via GeneCards.

Haplotype chr: SNP^a^	GeneHancer (GH) Identifier	GH Type	GH Score	GH Sources	Gene Association Score	ENCODE transcription factor (TF) binding sites	Gene Targets^c^	Cells and tissues^d^	RoadMap Epigenomics^e^
chr1: rs11368313	GH01J019798	Promoter/Enhancer	1.9^b^	RefSeq, EPDnew, Ensembl, ENCODE, CraniofacialAtlas	260.60^b^	110 TFs	6 genes; TMCO4	B-cells, immune cells, many other cells and tissues	B-cells, immune cells, many other cells
	GH01J019812	Promoter/Enhancer	1.7^b^	RefSeq, EPDnew, Ensembl, ENCODE, dbSUPER	259.30^b^	49 TFs	7 genes; TMCO4, RNF186, OTUD3		
chr4: rs10006825	GH04J026131	Enhancer	1.1^b^	FANTOM5, Ensembl, ENCODE	17.60^b^	46 TFs	6 genes; RBPJ	Immune cells	B-cells, T-cells, natural killer cells
chr6: rs10658790	GH06J125955	Promoter/Enhancer	2.6^b^	RefSeq, EPDnew, FANTOM5, Ensembl, ENCODE, CraniofacialAtlas, dbSUPER	250.70^b^	313 TFs	7 genes; HINT3, TRMT11	B-cells, immune cells, many other cells and tissues	B-cells, other immune cells, many other cells
chr10: rs10567579	GH10J132962	Promoter/Enhancer	1.4	RefSeq, Ensembl, ENCODE, CraniofacialAtlas	250.70^b^	20 TFs	7 genes; LINC01168, LINC01166	B-cells, immune cells, many other cells and tissues	B-cells, other immune cells, many other cells
	GH10J133008	Enhancer	1.3^b^	RefSeq, ENCODE, CraniofacialAtlas, dbSUPER	29.7	43 TFs	12 genes; LINC01168	Some immune cells	Monocytes, neutrophils, natural killer cells
	GH10J133012	Enhancer	1.3^b^	RefSeq, FANTOM5, ENCODE, dbSUPER	28.1	15 TFs	8 genes; LINC01168	Monocytes	Monocytes, neutrophiles, natural killer cells
chr11: rs106282082	GH11J134729	Enhancer	0.8^b^	RefSeq, ENCODE, dbSUPER	256.80^b^	4 TFs	5 genes; LINC02714		
chr17: rs111362005	GH17J000914	Enhancer	1.5^b^	RefSeq, FANTOM5, Ensembl, ENCODE, CraniofacialAtlas, dbSUPER	6.40^b^	17 TFs	7 genes; NXN	Neutrophils, some other cells and tissues	Neutrophils, some other cells
	GH17J000924	Promoter/Enhancer	1.8^b^	RefSeq, Ensembl, ENCODE, CraniofacialAtlas, dbSUPER	17.90^b^	162 TFs	12 genes; NXN	Some cells and tissues	Some cells
	GH17J000931	Promoter/Enhancer	1.6^b^	Ensembl, ENCODE, CraniofacialAtlas, dbSUPER	29.60^b^	122 TFs	12 genes; NXN	Some cells and tissues	
chr17: rs1024819	GH17J057521	Enhancer	1.6^b^	RefSeq, Ensembl, ENCODE, CraniofacialAtlas, dbSUPER	0.06	114TFs	9 genes; MSI2	B-cells, immune cells; SE_13320 (CD34 primary cells); many other cells and tissues	B-cells, some other immune cells, some other cells
chr17: rs10163481	GH17J077084	Enhancer	0.4^b^	Ensembl, ENCODE, dbSUPER	255.60^b^	no	6 genes; SNHG20		
	GH17J077087	Promoter/Enhancer	2.5^b^	RefSeq, EPDnew, FANTOM5, Ensembl, ENCODE, CraniofacialAtlas, dbSUPER	250.70^b^	263TFs	10 genes; SEC14L1, SNHG20	B-cells, immune cells	B-cells, other immune cells, many other cells
	GH17J077095	Enhancer	1.3^b^	FANTOM5, Ensembl, ENCODE, dbSUPER	26.60^b^	83 TFs	8 genes; SEC14L1, SNHG20	Immune cells	Immune cells, many other cells
	GH17J077106	Promoter/Enhancer	1.9^b^	RefSeq, FANTOM5, Ensembl, ENCODE, CraniofacialAtlas, dbSUPER	18.50^b^	121 TFs	10 genes; SEC14L1	B-cells, lymphocyte of B lineage, immune cells	B-cells, other immune cells, many other cells
	GH17J077114	Promoter/Enhancer	2.0^b^	RefSeq, FANTOM5, Ensembl, ENCODE, CraniofacialAtlas, dbSUPER	10.20^b^	233 TFs	9 genes; SEC14L1	B-cells, immune cells	B-cells, immune cells, many other cells
	GH17J077137	Promoter/Enhancer	2.6^b^	RefSeq, EPDnew, FANTOM5, Ensembl, ENCODE, CraniofacialAtlas, dbSUPER	259.8^b^	300 TFs	12 genes; SEC14L1	B-cells, immune cells, other cells	B-cells, immune cells, many other cells
	GH17J077150	Enhancer	0.3^b^	ENCODE, dbSUPER	7.20^b^	no	7 genes; SEC14L1		
	GH17J077152	Enhancer	0.3^b^	ENCODE, dbSUPER	7.20^b^	no	6 genes; SEC14L1		
	GH17J077159	Promoter/Enhancer	1.2^b^	RefSeq, ENCODE, dbSUPER	7.20^b^	54 TFs	7 genes; SEC14L1	B-cells	B-cells, immune cells, some other cells
chr19: rs10420324	GH19J053864	Promoter/Enhancer	2.6^b^	RefSeq, EPDnew, FANTOM5, Ensembl, ENCODE, CraniofacialAtlas, dbSUPER	265.10^b^	255 TFs	39 genes; MYADM, ZNF765, ZNF813, ZNF761, CNOT3, PRKCG	B-cells, immune cells; SE_20401 (CD56 primary cells), SE in many immune cells	B-cells, immune cells, many other cells
	GH19J053874	Enhancer	0.6^b^	ENCODE, dbSUPER	11.00^b^	5 TFs	6 genes; MYADM	B-cells, immune cells; SE_20401 (CD56 primary cells), SE in many immune cells	B-cells, immune cells
	GH19J053877	Enhancer	0.6^b^	ENCODE, dbSUPER	6.90^b^	5 TFs	10 genes; PRKCG	B-cells; SE in some immune cells	Many cells
	GH19J053889	Enhancer	0.8^b^	ENCODE, dbSUPER	18.50^b^	24 TFs	6 genes; MYADM	Immune cells	B-cells, monocytes, natural killer cells
	GH19J053907	Promoter/Enhancer	1.2^b^	FANTOM5, Ensembl, ENCODE	260.40^b^	31 TFs	8 genes; CACNG7, PRKCG, CACNG8		
	GH19J053956	Promoter/Enhancer	1.8^b^	RefSeq, EPDnew, FANTOM5, Ensembl, ENCODE	260.40^b^	54 TFs	23 genes; CACNG8, CACNG7		
	GH19J053977	Promoter/Enhancer	1.5^b^	RefSeq, Ensembl, ENCODE	18.50^b^	149 TFs	27 genes; CACNG8	B-cells, immune cells	Some cells
chr20: rs1051904	GH20J004133	Enhancer	1.7^b^	efSeq, FANTOM5, Ensembl, ENCODE, dbSUPER	3.00^b^	149TFs	10 genes; SMOX	Monocytes, neutrophils, other cells and tissues	Some cells
	GH20J004148	Promoter/Enhancer	2.0^b^	RefSeq, EPDnew, Ensembl, ENCODE, CraniofacialAtlas, dbSUPER	268.70^b^	83 TFs	6 genes; SMOX		Immune cells, many other cells
	GH20J004159	Enhancer	1.2^b^	RefSeq, FANTOM5, ENCODE, dbSUPER	8.60^b^	20 TFs	6 genes; SMOX	Monocytes	B-cells, other immune cells, many other cells
	GH20J004165	Enhancer	1.5^b^	RefSeq, FANTOM5, ENCODE, CraniofacialAtlas, dbSUPER	20.7^b^	36 TFs	9 genes; SMOX		Some cells
	GH20J004169	Promoter/Enhancer	2.1^b^	RefSeq, Ensembl, ENCODE, CraniofacialAtlas, dbSUPER	7.90^b^	190 TFs	7 genes; SMOX	B-cells, immune cells	B-cells, other immune cells, many other cells
chr20: rs111797554	GH20J057617	Promoter/Enhancer	2.0^b^	RefSeq, EPDnew, FANTOM5, Ensembl, ENCODE, dbSUPER	264.30^b^	67 TFs	4 genes; ZBP1	B-cells, immune cells; SE_20522 (CD56 primary cells), SE_16712 (CD4 naive primary cells)	B-cells, immune cells, gastrointestinal tract
	GH20J057627	Enhancer	1.1^b^	FANTOM5, ENCODE, CraniofacialAtlas, dbSUPER	11.90^b^	13 TFs	8 genes; ZBP1	B-cells, immune cells; SE_20522 (CD56 primary cells), SE_16712 (CD4 naive primary cells)	B-cells, other immune cells, many other cells
chr 22: rs1045588	GH22J017767	Enhancer	1.2^b^	RefSeq, ENCODE, dbSUPER	4.90^b^	49 TFs	4 genes; BID	Immune cells; SE_09461 (CD14+ monocytes)	B-cells, other immune cells, some other cells
	GH22J017769	Promoter/Enhancer	2.5^b^	RefSeq, EPDnew, FANTOM5, Ensembl, ENCODE, CraniofacialAtlas, dbSUPER	264.80^b^	155 TFs	7 genes; BID	B-cells, immune cells; SE_09461 (CD14+ monocytes); many cells and tissues	B-cells, other immune cells, many other cells
	GH22J017784	Enhancer	1.8^b^	RefSeq, VISTA, ENCODE, CraniofacialAtlas, dbSUPER	0.41	153 TFs	8 genes	B-cells, immune cells; SE_09461 (CD14+ monocytes)	Immune cells, many other cells
	GH22J017794	Enhancer	1.6^b^	RefSeq, FANTOM5, Ensembl, ENCODE, dbSUPER	15.80^b^	83 TFs	10 genes; BID, MICAL3	B-cells, immune cells; SE_09461 (CD14+ monocytes)	B-cells, monocytes, hematopoietic stem cells, natural killer cells

^a^Haplotype identified by the chromosome and the joint SNP in the haplotype region.

^b^Double Elite GeneHancer score and Gene association score.

^c^Number of gene targets; Gene targets in the haplotype region.

^d^Examples of tissues, in which the promoter/enhancer is active in GeneHancer sources; Superenhancer (SE) in B-cells or immune cells.

^e^Cell populations with active chromatin state in 6 immune cell populations and over 10 adult cell and tissue types were extracted via Roadmap Epigenomics.

**Fig. 2:**
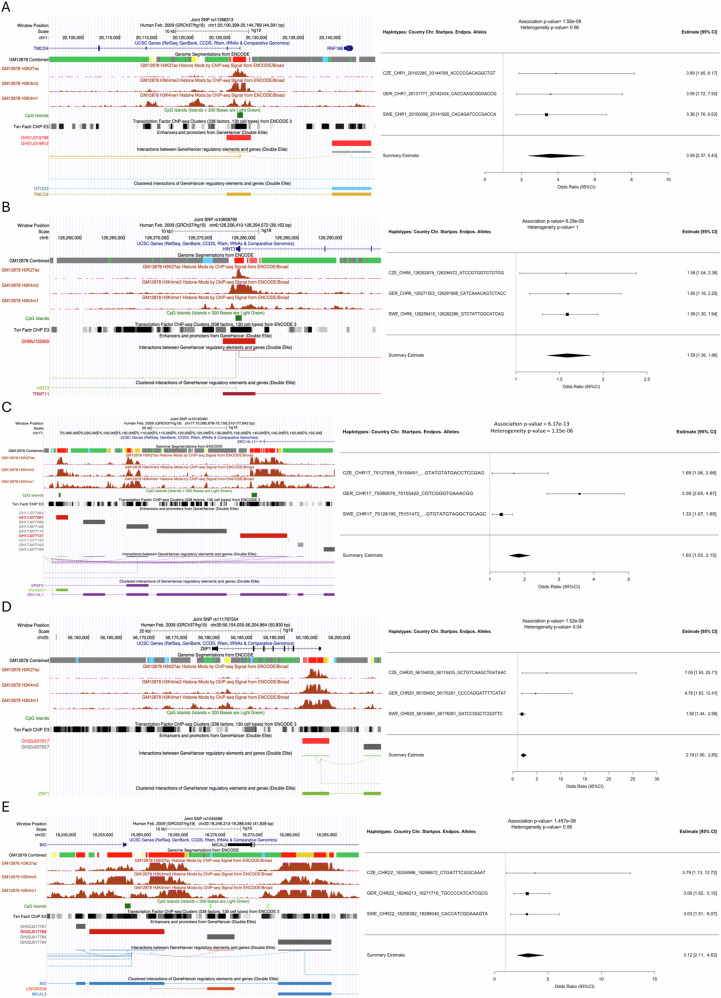
Haplotype regions and Forest plots from the five most interesting haplotypes associated with the risk of MGUS. Haplotype regions are shown using UCSC Genome browser’s GRCh37/hg19 assembly and annotation tracks from ENCODE and GeneHancer. Forest plots show the overlapping haplotypes, represented by a joint SNP, of the three study populations from the Czech Republic (CZE), Germany (GER) and Sweden (SWE). For each population the odds ratio and the corresponding 95% confidence interval (CI) are shown as well as the summary estimate of the meta-analysis. **A** Chromosome 1, rs11368313, **B** Chromosome 6, rs10658790, **C** Chromosome 17, rs10163481, **D** Chromosome 20, rs111797554, **E** Chromosome 22, rs1045588.

In five haplotype regions, strong promoter, enhancer and regulatory element related histone marks and regulatory element-target gene association scores as well as ChrommHMM and Segway genome segmentation data supported the importance of the regions in MGUS susceptibility (Fig. 2, Table 2). On chromosome 1, the region represented by rs11368313 overlapped with *TMCO4* (transmembrane and coiled-coil domains 2) and *RNF186* (ring finger protein 186) and showed interactions between promoter/enhancers of these two genes. Additionally, *RNF186* interacted with *OTUD3* (OTU deubiquitinase 3). Both RNF186 and OTUD3 are involved in (de)ubiquitination. A haplotype on chromosome 6 represented by rs10658790 overlapped with the promoter/enhancer of *HINT3* (histidine triad nucleotide binding protein 3), which interacted with promoter/enhancers of *TRMT11* (tRNA methyltransferase 11 homolog) and *NCOA7* (Nuclear Receptor Coactivator 7). HINT3 may be involved in PI3K/AKT/mTOR pathway.

On chromosome 17, the most interesting haplotype region represented by rs10163481 contained three regions with strong promoter and enhancer histone marks (Fig. 2, Table 2). These were related to GeneHancer promoter/enhancers and showed interactions between *SEC14L1* (SEC14 like lipid binding 1) and *SNHG20* (small nucleolar RNA host gene 20) and *SRSF2* (serine and arginine rich splicing factor 2) which is involved in RNA splicing. *SEC14L1* encodes a signal transduction inhibitor involved in innate immunity.

The region on chromosome 20 represented by rs111797554 overlapped with the *ZBP1* (Z-DNA binding protein 1) gene and its promoter and enhancer that interact with each other (Fig. 2, Table 2). ZBP1 is involved in innate immunity responses. The region on chromosome 22, represented by rs1045588, involved two genes, *BID* (BH3 interacting domain death agonist) and *MICAL3* (microtubule associated monooxygenase, calponin and LIM domain containing 3), and showed several regions with very strong promoter and enhancer histone marks and promoter-enhancer interactions. BID belongs to the BCL-2 family of cell death regulators and MICAL3 is involved in cell cycle regulation.

Further five haplotype regions with either weaker histone marks, genome segmentation data or regulatory element-target gene associations included a region on chromosome 4 represented by rs10006825 which contained an enhancer associated with *RBPJ* (recombination signal binding protein for immunoglobulin kappa J region) (Table 2, Supplementary Fig. 1). RBPJ is regulating NOTCH-signaling. In the region on chromosome 17 represented by rs111362005 three enhancers within an intron of *NXN* (nucleoredoxin) showed interactions with an enhancer region flanking the promoter of *NXN*, which regulates Wnt signaling. In another region on chromosome 17, represented by rs1024819, a weak enhancer interaction within *MSI2* (musashi RNA binding protein 2) may affect the function of MSI2 as a transcriptional regulator of genes involved in development and cell cycle.

A large region on chromosome 19 represented by rs10420324 hosted *MYADM* (myeloid associated differentiation marker), *PRKCG* (protein kinase C gamma), *CACNG7* and *CACNG8* (calcium voltage-gated channel auxiliary subunit gamma 7 and 8, respectively) that interacted with each other as well as with three zink finger proteins and *CNO3* (CCR4-NOT Transcription Complex Subunit 3), which is involved in RNA-mediated gene silencing (Table 2, Supplementary Fig. 1). MYADM is a negative regulator of heterotypic cell-cell adhesion and protein kinase C signaling. On chromosome 20 represented by rs1051904 many promoter-enhancer interactions were implicated between the *SMOX* (spermine oxidase) gene and long-distance enhancers. SMOX may act as a determinant of cellular sensitivity to antitumor polyamine analogs.

Furthermore, on chromosomes 10 and 11, GeneHancer regulatory elements were connected to long non-coding RNAs, however with unknown functional consequences (Table 2, Supplementary Fig. 1). On chromosome 12, a region represented by rs10840622 overlapped with the *SETD1B* (SET Domain Containing 1B, Histone Lysine Methyltransferase) and *HPD* (4-Hydroxyphenylpyruvate Dioxygenase) genes and showed strong promoter-related histone marks at the 3’end of *HPD*, but no interactions with any GeneHancer regulatory elements.

## Discussion

We conducted a haplotype-based analysis on MGUS to complement the earlier GWAS analyses where individual SNPs were considered [13–15, 29]. A previous meta-analysis of the GWAS data from MGUS, MM and AL-amyloidosis identified 17 independent regions with genome-wide significance [13]. In the present study we identified 23 haplotypes that were associated with the risk of MGUS at the genome-wide significance level *p* < 5 × 10^−8^ and showed increased risk of MGUS in all three study populations. Notably, only the *ULK4* containing haplotype on chromosome 3 shared significance (OR 1.70, *p* = 1.13 × 10^−8^) with the previous study, which may suggest the importance of this locus. ULK4 is a serine/threonine kinase. Several associated haplotype regions affected pathways important for MM cell survival and genes encoding important current therapeutic targets for MM, which may highlight the advantage of the haplotype approach homing to functional units.

We identified five haplotype regions, in which associations with regulatory elements and their connections to target genes supported by genome segmentation highlighted the possible role of the related haplotypes in MGUS. None of these regulatory elements were specific for B-cells, but were active also in other immune cells, and other adult cells and tissues, as implicated from different sources from GeneHancer and Roadmap Epigenomics. The haplotype on chromosome 1 included two (de)ubiquitination-related genes, *RNF186* and *OTUD3*, which interacted with each other. As MM cells produce high amounts of monoclonal antibodies, maintaining protein homeostasis is crucial for MM cells [30]. The ubiquitin–proteasome system plays an important role in this process and MM cell killing can be caused by blocking or interfering this system. Thus, it has been one of the primary targets in MM treatment starting in early 2000 with proteasome inhibitors and immune modulators [30]. Commonly used proteasome inhibitors include bortezomib, carfilzomib and the oral drug ixazomib [31]. Commonly used immune modulators include thalidomide, lenalidomide and pomalidomide [31]. E3 ubiquitin ligase and deubiquitination inhibitors are currently being tested in preclinical studies and clinical trials [30, 31].

A haplotype on chromosome 6 covered the promoter/enhancer of *HINT3*, probably involved in PI3K/AKT/mTOR pathway, and interacting with promoter/enhancers of *NCOA7* which encodes an estrogen receptor associated protein. PI3K/AKT/mTOR pathway is one of the signaling pathways in the bone marrow microenvironment that promotes signaling events in MM cells and enhances their survival [32]. The pathway shows aberrant activation in many MM patients. Targeting PI3K/AKT/mTOR pathway has shown promising results in preclinical studies, however, clinical trials have been disappointing showing limited clinical efficiency in MM and severe side effects [32].

The haplotype on chromosome 17 encoding the *SEC14L1* gene and another haplotype on chromosome 20 associated with the *ZBP1* gene both are involved in innate immunity. *SEC14L1* was one of the genes that was mutated in the germline of one German MM family [33]. ZBP1 is integral part of host defense against pathogens. It responds to a variety of stimuli, such as viral infection and homeostatic perturbations, leading to the formation of the PANoptosome complex and release of numerous cytokines and chemokines [34]. It is also known to be active in MM [35].

The haplotype on chromosome 22 covered two genes, *BID* and *MICAL3*. MICAL3 is involved in cell cycle regulation and it has been implicated in various cancers [36]. BID belongs to the BCL-2 family of cell death regulators which have recently become extremely promising therapeutic targets in hematological malignancies, including MM, with the prime drug venetoclax [31, 37, 38]. Overexpression of Bcl-2 has been found in MM harboring a common translocation (11;14) which is a special indication for venetoclax [39]. This translocation is also common in MGUS [40]. Unfortunately, the MGUS samples of the present study have not been screened for cytogenetic alterations and we could not investigate this aspect further.

A further haplotype region with a possible association with immune functions was found on chromosome 4 represented with an enhancer associated with *RBPJ*. This enhancer seemed to be specific for immune cells, especially for B-cells, T-cells and natural killer cells. RBPJ is a key translational transducer of NOTCH-signaling and it plays a role in cellular immune response [41]. Similar to PI3K/AKT/mTOR pathway, NOTCH-signaling promotes communication between adjacent cells in the bone marrow microenvironment and has been shown to be dysregulated in the MM tumor niche [42].

The challenges of our haplotype-based association study included three different study populations and their different sample sizes as well as different genotyping arrays used for the sample sets. This also illustrates some limitations of our study. In general, use of several populations increases the sample size and allows replication of the findings. However, as the frequencies of the SNPs and their LD vary between populations, it is difficult to find exactly the same haplotypes in all populations. The use of different genotyping arrays with different numbers of genotyped SNPs affected the imputation and the final number of SNPs included in the study, and at the end the composition of the haplotypes. We aimed to solve these problems by identifying the common SNPs in all populations and defining a joint SNP as a representative for the haplotype as shown in Fig. 1. However, the different composition of the haplotypes in the study populations makes it also difficult to evaluate the effect of the haplotypes on the expression of genes connected to these haplotypes. We used GeneHancer and Roadmap Epigenomics data to investigate the activity of the regulatory elements in B-cells, other immune cells and other adult cells and tissues as a potential indicator for the effect of the haplotypes. The problem of different sample sizes in the meta-analysis of GWAS data has been considered by Cook et al. [43]. They demonstrated that linear/logistic models can be used for meta-analysis of GWASs of binary phenotypes, without loss of power, even in the presence of extreme sample size and case–control imbalances, provided that inverse-variance weighting of allelic effect sizes after conversion onto the log-odds scale has been performed, as was done in our study.

In conclusion, our haplotype-based genetic association study identified several novel loci associated with the risk of MGUS. Genes and regulatory elements connected to neighboring genes were related to pathways dysregulated in MM, which serve as targets for already existing therapies, or may serve as targets for drugs that are currently tested in preclinical studies or clinical trials. Interestingly, these loci with exception of the *ULK4* locus, have not been found in large GWASs on MM which may show the strength of the haplotype-based approach testing functional chromosomal units rather than individual SNPs. Whether these MGUS-associated loci are important in the disease progression to MM as high-risk markers remains to be investigated.

### Supplementary information

Supplementary information

## Data Availability

The GWAS data are available at the NHGRI-EBI Catalog of human genome-wide association studies, accession number GCST007824. The datasets generated and/or analyzed during the current study are available from the corresponding author on reasonable request.
