# Supplementary material for: Haplotype analysis identifies functional elements in monoclonal gammopathy of unknown significance
Source: Blood Cancer J. 2024 Aug 20;14(1):140. doi: 10.1038/s41408-024-01121-8 (PMC11335940; doi:10.1038/s41408-024-01121-8)
Supplement: Supplementary file 1 — Supplementary information [file 41408_2024_1121_MOESM1_ESM.pdf]

HAPLOTYPE ANALYSIS IDENTIFIES FUNCTIONAL ELEMENTS IN MONOCLONAL GAMMOPATHY OF UNKNOWN SIGNIFICANCE

Running title: Haplotype analysis identifies functional elements

Hauke Thomsen[1], Subhayan Chattopadhyay[2], Niels Weinhold[3], Pavel Vodicka[4,5,6], Ludmila Vodickova[4,5,6], Per Hoffmann[7,8], Markus M Nöthen[7], Karl-Heinz Jöckel[9], Börge Schmidt[9], Roman Hajek[10], Göran Hallmans[11], Ulrika Pettersson-Kymmer[12], Florentin Späth[13], Hartmut Goldschmidt[3,14], Kari Hemminki[6,15], Asta Försti[16,17]

SUPPLEMENTARY INFORMATION

Supplementary material includes 1 figure showing the UCSC Genome browser plots and forest plots for the haplotype regions described in main text of the manuscript.

Supplementary Figure 1. Haplotype regions and Forest plots from 8 haplotypes associated with the risk of MGUS. The UCSC plots are shown using UCSC Genome browser's GRCh37/hg19 assembly and include tracks of UCSC genes, Genome segmentations from ENCODE, Histone modifications in the lymphoblastoid cell line GM12878 from ENCODE/Broad, CpG Islands, Transcription factor binding sites from ENCODE, Enhancers and promoters from GeneHancer (Double Elite) and Interactions between GeneHancer regulatory elements and genes (Double Elite). Forest plots show the overlapping haplotypes, represented by a joint SNP, of the three study populations from the Czech Republic (CZE), Germany (GER) and Sweden (SWE) with the chromosome, start and end position and the alleles of each haplotype. For each population the odds ratio and the corresponding 95% confidence interval (CI) are shown as well as the summary estimate of the meta-analysis. (A) Chromosome 4, rs10006825, (B) Chromosome 17, rs111362005, (C) Chromosome 17, rs1024819, (D) Chromosome 19, rs10420324, (E) Chromosome 20, rs1051904, (F) Chromosome 10, rs10567579, (G) Chromosome 11, rs10628082, (H) Chromosome 12, rs10840622.

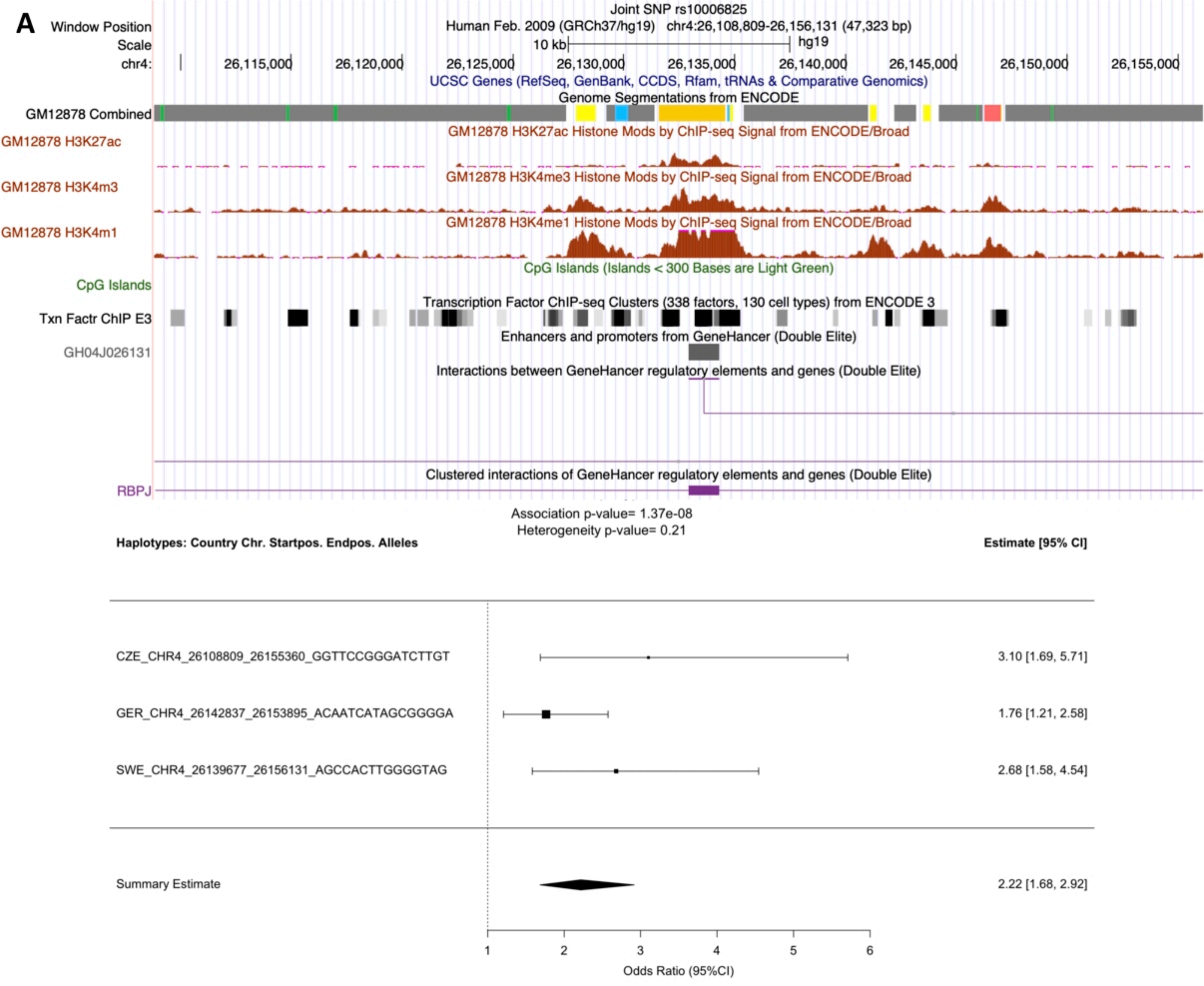
A
Window Position
Joint SNP rs10006825
Human Feb. 2009 (GRCh37/hg19) chr4:26,108,809-26,156,131 (47,323 bp)
Scale
10 kb
hg19
chr4:
26,115,000
26,120,000
26,125,000
26,130,000
26,135,000
26,140,000
26,145,000
26,150,000
26,155,000
UCSC Genes (RefSeq, GenBank, CCDS, Rfam, tRNAs & Comparative Genomics)
Genome Segmentations from ENCODE
GM12878 Combined
GM12878 H3K27ac
GM12878 H3K27ac Histone Mods by ChIP-seq Signal from ENCODE/Broad
GM12878 H3K4m3
GM12878 H3K4me3 Histone Mods by ChIP-seq Signal from ENCODE/Broad
GM12878 H3K4m1
GM12878 H3K4me1 Histone Mods by ChIP-seq Signal from ENCODE/Broad
CpG Islands (Islands < 300 Bases are Light Green)
CpG Islands
Transcription Factor ChIP-seq Clusters (338 factors, 130 cell types) from ENCODE 3
Txn Factr ChIP E3
Enhancers and promoters from GeneHancer (Double Elite)
GH04J026131
Interactions between GeneHancer regulatory elements and genes (Double Elite)
Clustered interactions of GeneHancer regulatory elements and genes (Double Elite)
RBPJ
Association p-value= 1.37e-08
Heterogeneity p-value= 0.21
Haplotypes: Country Chr. Startpos. Endpos. Alleles
Estimate [95% CI]
CZE_CHR4_26108809_26155360_GGTTCCGGGATCTTGT
3.10 [1.69, 5.71]
GER_CHR4_26142837_26153895_ACAATCATAGCGGGGA
1.76 [1.21, 2.58]
SWE_CHR4_26139677_26156131_AGCCACTTGGGGTAG
2.68 [1.58, 4.54]
Summary Estimate
2.22 [1.68, 2.92]
1
2
3
4
5
6
Odds Ratio (95%CI)

B

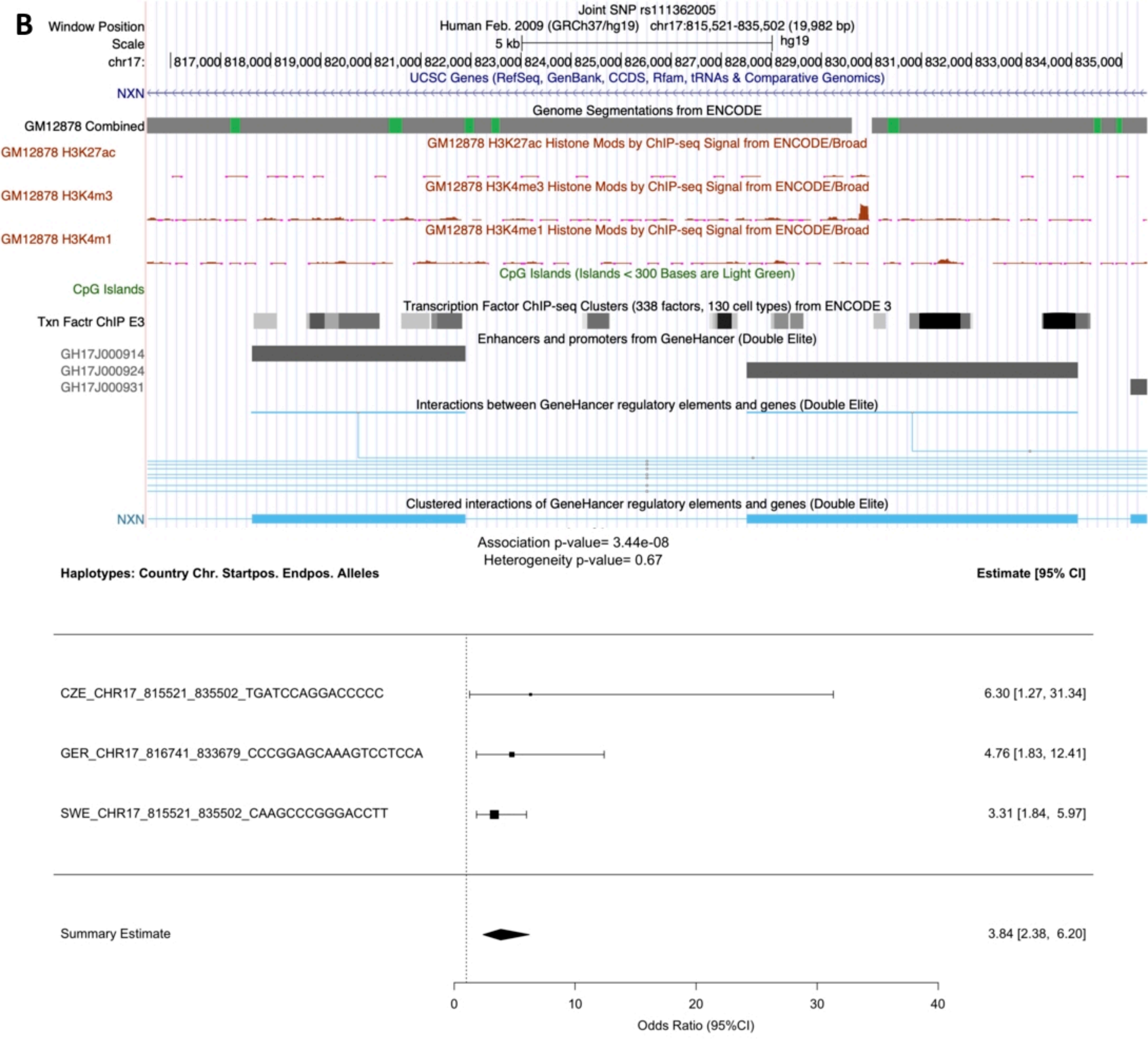
Window Position
Scale
chr17:
Joint SNP rs111362005
Human Feb. 2009 (GRCh37/hg19) chr17:815,521-835,502 (19,982 bp)
5 kb
hg19
817,000 818,000 819,000 820,000 821,000 822,000 823,000 824,000 825,000 826,000 827,000 828,000 829,000 830,000 831,000 832,000 833,000 834,000 835,000
UCSC Genes (RefSeq, GenBank, CCDS, Rfam, tRNAs & Comparative Genomics)
NXN
Genome Segmentations from ENCODE
GM12878 Combined
GM12878 H3K27ac
GM12878 H3K27ac Histone Mods by ChIP-seq Signal from ENCODE/Broad
GM12878 H3K4m3
GM12878 H3K4me3 Histone Mods by ChIP-seq Signal from ENCODE/Broad
GM12878 H3K4m1
GM12878 H3K4me1 Histone Mods by ChIP-seq Signal from ENCODE/Broad
CpG Islands (Islands < 300 Bases are Light Green)
CpG Islands
Transcription Factor ChIP-seq Clusters (338 factors, 130 cell types) from ENCODE 3
Txn Factr ChIP E3
Enhancers and promoters from GeneHancer (Double Elite)
GH17J000914
GH17J000924
GH17J000931
Interactions between GeneHancer regulatory elements and genes (Double Elite)
Clustered interactions of GeneHancer regulatory elements and genes (Double Elite)
NXN
Association p-value= 3.44e-08
Heterogeneity p-value= 0.67
Haplotypes: Country Chr. Startpos. Endpos. Alleles
Estimate [95% CI]
CZE_CHR17_815521_835502_TGATCCAGGACCCCC
6.30 [1.27, 31.34]
GER_CHR17_816741_833679_CCCGGAGCAAAGTCCTCCA
4.76 [1.83, 12.41]
SWE_CHR17_815521_835502_CAAGCCCGGGACCTT
3.31 [1.84, 5.97]
Summary Estimate
3.84 [2.38, 6.20]
0
10
20
30
40
Odds Ratio (95%CI)

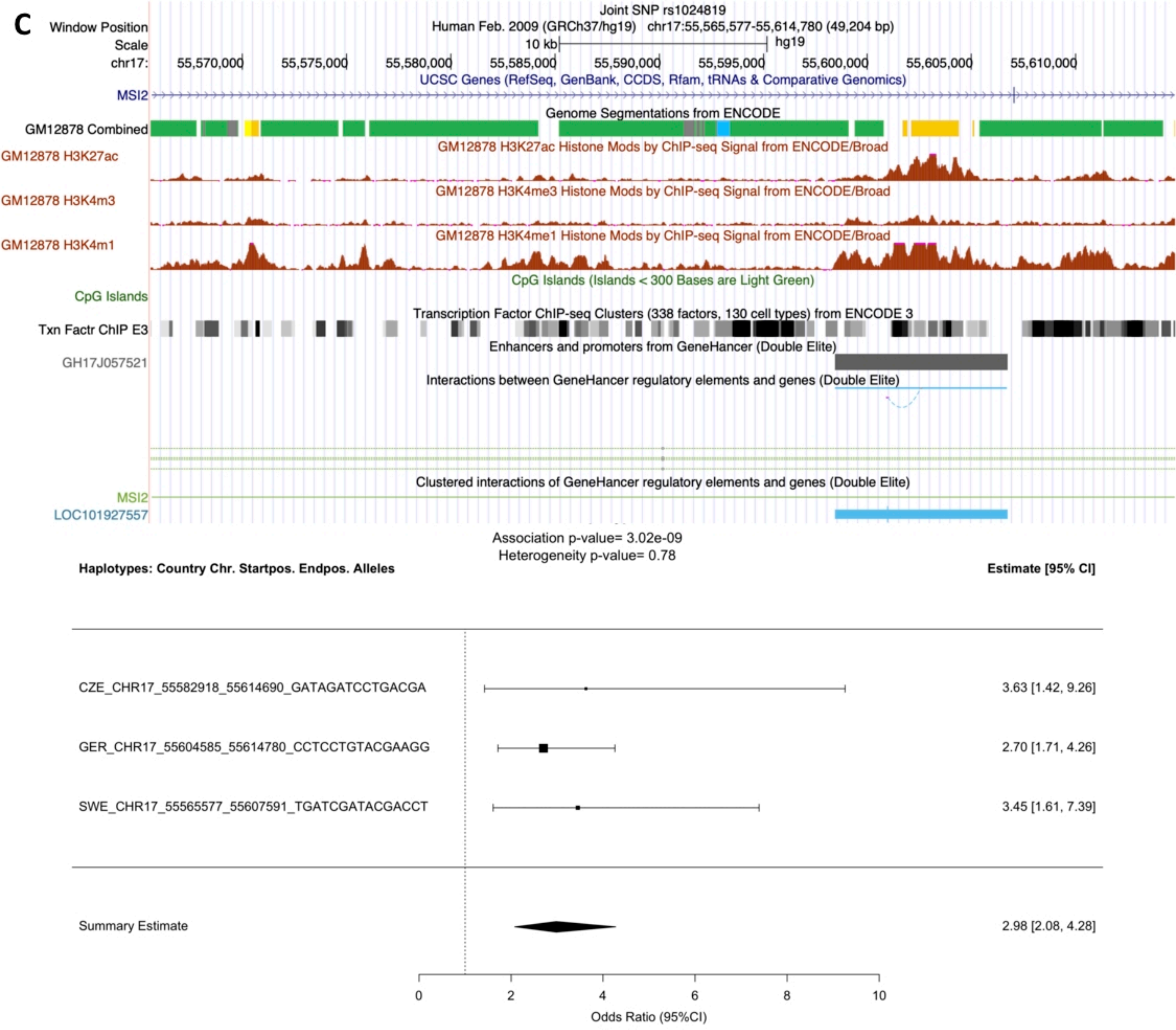
C
Window Position
Human Feb. 2009 (GRCh37/hg19) chr17:55,565,577-55,614,780 (49,204 bp)
Joint SNP rs1024819
Scale
10 kb
hg19
chr17:
55,570,000
55,575,000
55,580,000
55,585,000
55,590,000
55,595,000
55,600,000
55,605,000
55,610,000
UCSC Genes (RefSeq, GenBank, CCDS, Rfam, tRNAs & Comparative Genomics)
MSI2
Genome Segmentations from ENCODE
GM12878 Combined
GM12878 H3K27ac Histone Mods by ChIP-seq Signal from ENCODE/Broad
GM12878 H3K27ac
GM12878 H3K4me3 Histone Mods by ChIP-seq Signal from ENCODE/Broad
GM12878 H3K4m3
GM12878 H3K4me1 Histone Mods by ChIP-seq Signal from ENCODE/Broad
GM12878 H3K4m1
CpG Islands (Islands < 300 Bases are Light Green)
CpG Islands
Transcription Factor ChIP-seq Clusters (338 factors, 130 cell types) from ENCODE 3
Txn Factr ChIP E3
Enhancers and promoters from GeneHancer (Double Elite)
GH17J057521
Interactions between GeneHancer regulatory elements and genes (Double Elite)
Clustered interactions of GeneHancer regulatory elements and genes (Double Elite)
MSI2
LOC101927557
Association p-value= 3.02e-09
Heterogeneity p-value= 0.78
Haplotypes: Country Chr. Startpos. Endpos. Alleles
Estimate [95% CI]
CZE_CHR17_55582918_55614690_GATAGATCCTGACGA
3.63 [1.42, 9.26]
GER_CHR17_55604585_55614780_CCTCCTGTACGAAGG
2.70 [1.71, 4.26]
SWE_CHR17_55565577_55607591_TGATCGATACGACCT
3.45 [1.61, 7.39]
Summary Estimate
2.98 [2.08, 4.28]
0
2
4
6
8
10
Odds Ratio (95%CI)

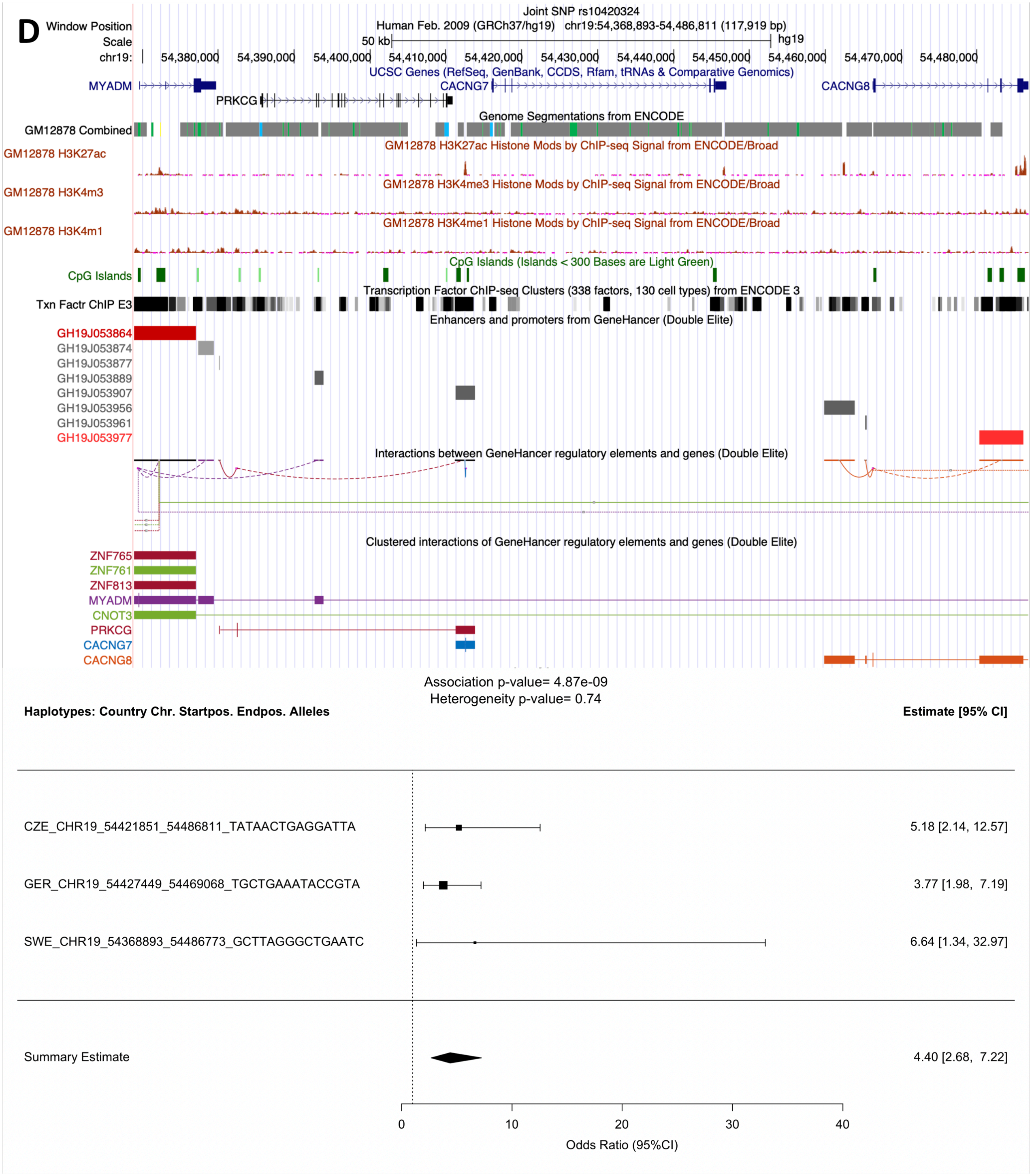
D
Window Position
Scale
chr19:
Joint SNP rs10420324
Human Feb. 2009 (GRCh37/hg19) chr19:54,368,893-54,486,811 (117,919 bp)
50 kb
hg19
54,380,000
54,390,000
54,400,000
54,410,000
54,420,000
54,430,000
54,440,000
54,450,000
54,460,000
54,470,000
54,480,000
UCSC Genes (RefSeq, GenBank, CCDS, Rfam, tRNAs & Comparative Genomics)
MYADM
PRKCG
CACNG7
CACNG8
Genome Segmentations from ENCODE
GM12878 Combined
GM12878 H3K27ac
GM12878 H3K27ac Histone Mods by ChIP-seq Signal from ENCODE/Broad
GM12878 H3K4m3
GM12878 H3K4me3 Histone Mods by ChIP-seq Signal from ENCODE/Broad
GM12878 H3K4m1
GM12878 H3K4me1 Histone Mods by ChIP-seq Signal from ENCODE/Broad
CpG Islands
CpG Islands (Islands < 300 Bases are Light Green)
Txn Factr ChIP E3
Transcription Factor ChIP-seq Clusters (338 factors, 130 cell types) from ENCODE 3
Enhancers and promoters from GeneHancer (Double Elite)
GH19J053864
GH19J053874
GH19J053877
GH19J053889
GH19J053907
GH19J053956
GH19J053961
GH19J053977
Interactions between GeneHancer regulatory elements and genes (Double Elite)
Clustered interactions of GeneHancer regulatory elements and genes (Double Elite)
ZNF765
ZNF761
ZNF813
MYADM
CNOT3
PRKCG
CACNG7
CACNG8
Association p-value= 4.87e-09
Heterogeneity p-value= 0.74
Haplotypes: Country Chr. Startpos. Endpos. Alleles
Estimate [95% CI]
CZE_CHR19_54421851_54486811_TATAACTGAGGATTA
5.18 [2.14, 12.57]
GER_CHR19_54427449_54469068_TGCTGAAATACCGTA
3.77 [1.98, 7.19]
SWE_CHR19_54368893_54486773_GCTTAGGGCTGAATC
6.64 [1.34, 32.97]
Summary Estimate
4.40 [2.68, 7.22]
0
10
20
30
40
Odds Ratio (95%CI)

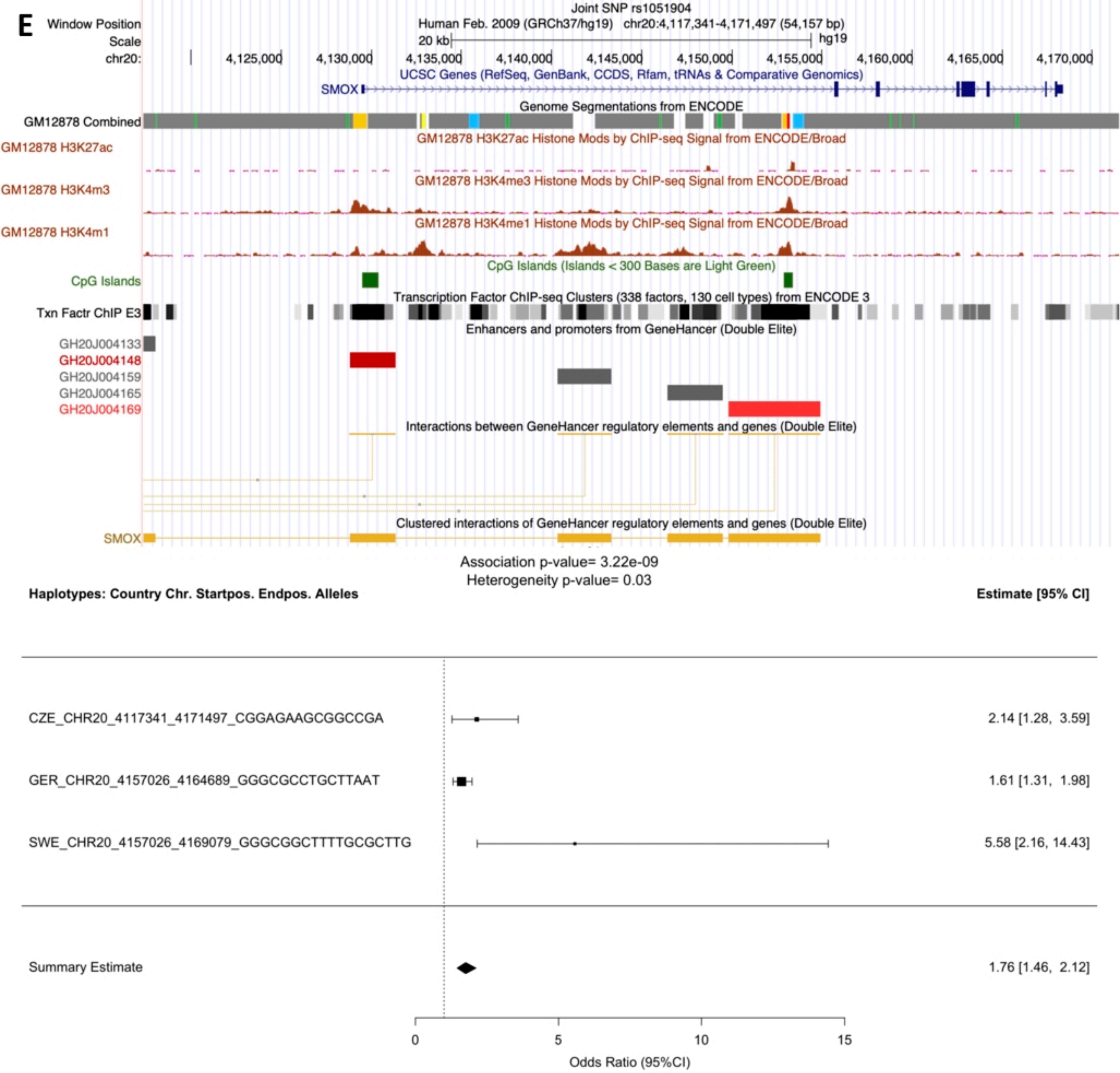
E
Joint SNP rs1051904
Window Position
Human Feb. 2009 (GRCh37/hg19) chr20:4,117,341-4,171,497 (54,157 bp)
Scale
20 kb
hg19
chr20:
4,125,000
4,130,000
4,135,000
4,140,000
4,145,000
4,150,000
4,155,000
4,160,000
4,165,000
4,170,000
UCSC Genes (RefSeq, GenBank, CCDS, Rfam, tRNAs & Comparative Genomics)
SMOX
Genome Segmentations from ENCODE
GM12878 Combined
GM12878 H3K27ac Histone Mods by ChIP-seq Signal from ENCODE/Broad
GM12878 H3K27ac
GM12878 H3K4me3 Histone Mods by ChIP-seq Signal from ENCODE/Broad
GM12878 H3K4m3
GM12878 H3K4me1 Histone Mods by ChIP-seq Signal from ENCODE/Broad
GM12878 H3K4m1
CpG Islands (Islands < 300 Bases are Light Green)
CpG Islands
Transcription Factor ChIP-seq Clusters (338 factors, 130 cell types) from ENCODE 3
Txn Factr ChIP E3
Enhancers and promoters from GeneHancer (Double Elite)
GH20J004133
GH20J004148
GH20J004159
GH20J004165
GH20J004169
Interactions between GeneHancer regulatory elements and genes (Double Elite)
Clustered interactions of GeneHancer regulatory elements and genes (Double Elite)
SMOX
Association p-value= 3.22e-09
Heterogeneity p-value= 0.03
Haplotypes: Country Chr. Startpos. Endpos. Alleles
Estimate [95% CI]
CZE_CHR20_4117341_4171497_CGGAGAAGCGGCCGA
2.14 [1.28, 3.59]
GER_CHR20_4157026_4164689_GGGCGCCTGCTTAAT
1.61 [1.31, 1.98]
SWE_CHR20_4157026_4169079_GGGCGGCTTTTGCGCTTG
5.58 [2.16, 14.43]
Summary Estimate
1.76 [1.46, 2.12]
0
5
10
15
Odds Ratio (95%CI)

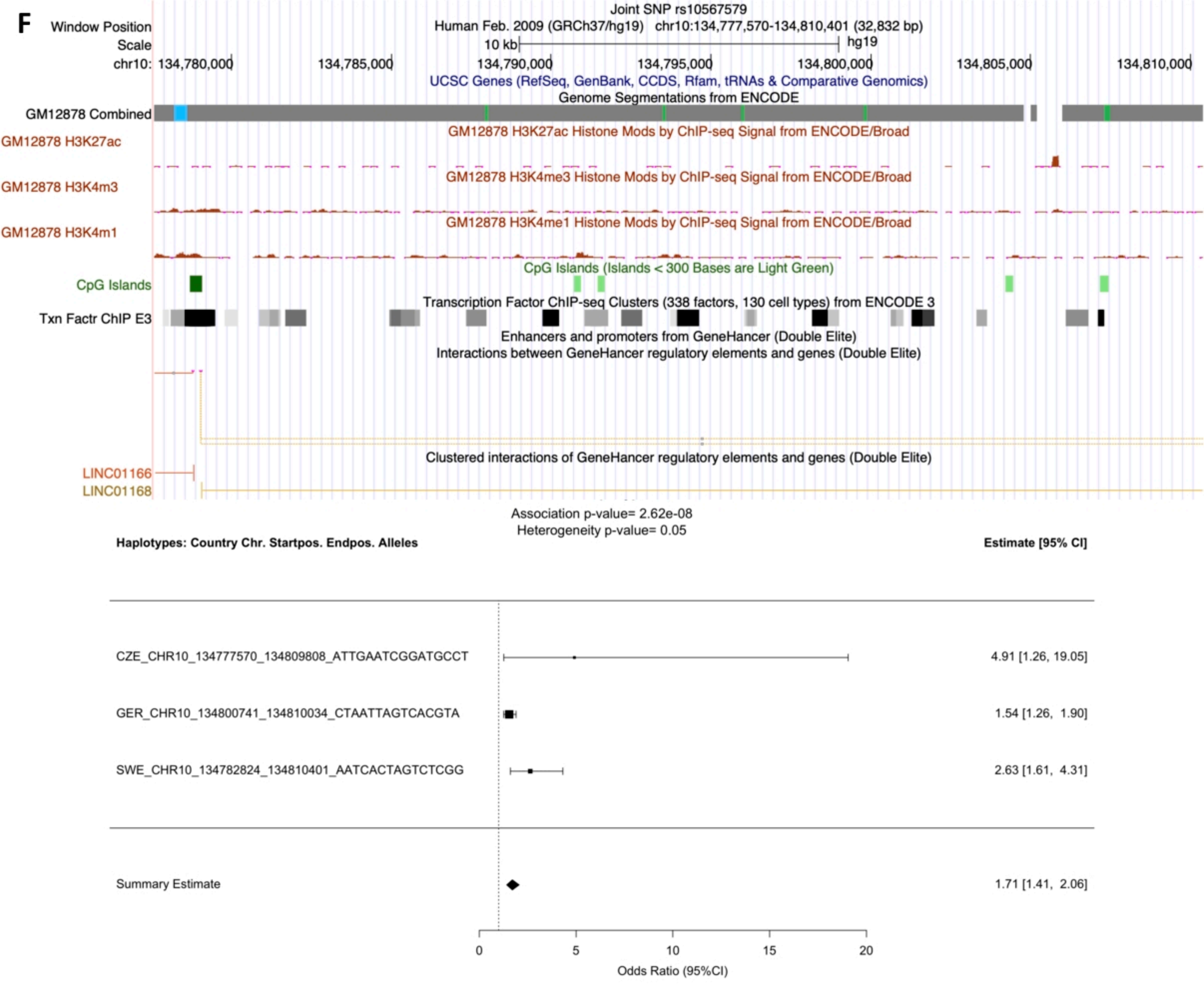
F
Joint SNP rs10567579
Window Position
Human Feb. 2009 (GRCh37/hg19) chr10:134,777,570-134,810,401 (32,832 bp)
Scale
10 kb
hg19
chr10: 134,780,000 134,785,000 134,790,000 134,795,000 134,800,000 134,805,000 134,810,000
UCSC Genes (RefSeq, GenBank, CCDS, Rfam, tRNAs & Comparative Genomics)
Genome Segmentations from ENCODE
GM12878 Combined
GM12878 H3K27ac Histone Mods by ChIP-seq Signal from ENCODE/Broad
GM12878 H3K27ac
GM12878 H3K4me3 Histone Mods by ChIP-seq Signal from ENCODE/Broad
GM12878 H3K4m3
GM12878 H3K4me1 Histone Mods by ChIP-seq Signal from ENCODE/Broad
GM12878 H3K4m1
CpG Islands (Islands < 300 Bases are Light Green)
CpG Islands
Transcription Factor ChIP-seq Clusters (338 factors, 130 cell types) from ENCODE 3
Txn Factr ChIP E3
Enhancers and promoters from GeneHancer (Double Elite)
Interactions between GeneHancer regulatory elements and genes (Double Elite)
Clustered interactions of GeneHancer regulatory elements and genes (Double Elite)
LINC01166
LINC01168
Association p-value= 2.62e-08
Heterogeneity p-value= 0.05
Haplotypes: Country Chr. Startpos. Endpos. Alleles
Estimate [95% CI]
CZE_CHR10_134777570_134809808_ATTGAATCGGATGCCT
4.91 [1.26, 19.05]
GER_CHR10_134800741_134810034_CTAATTAGTCACGTA
1.54 [1.26, 1.90]
SWE_CHR10_134782824_134810401_AATCACTAGTCTCGG
2.63 [1.61, 4.31]
Summary Estimate
1.71 [1.41, 2.06]
0
5
10
15
20
Odds Ratio (95%CI)

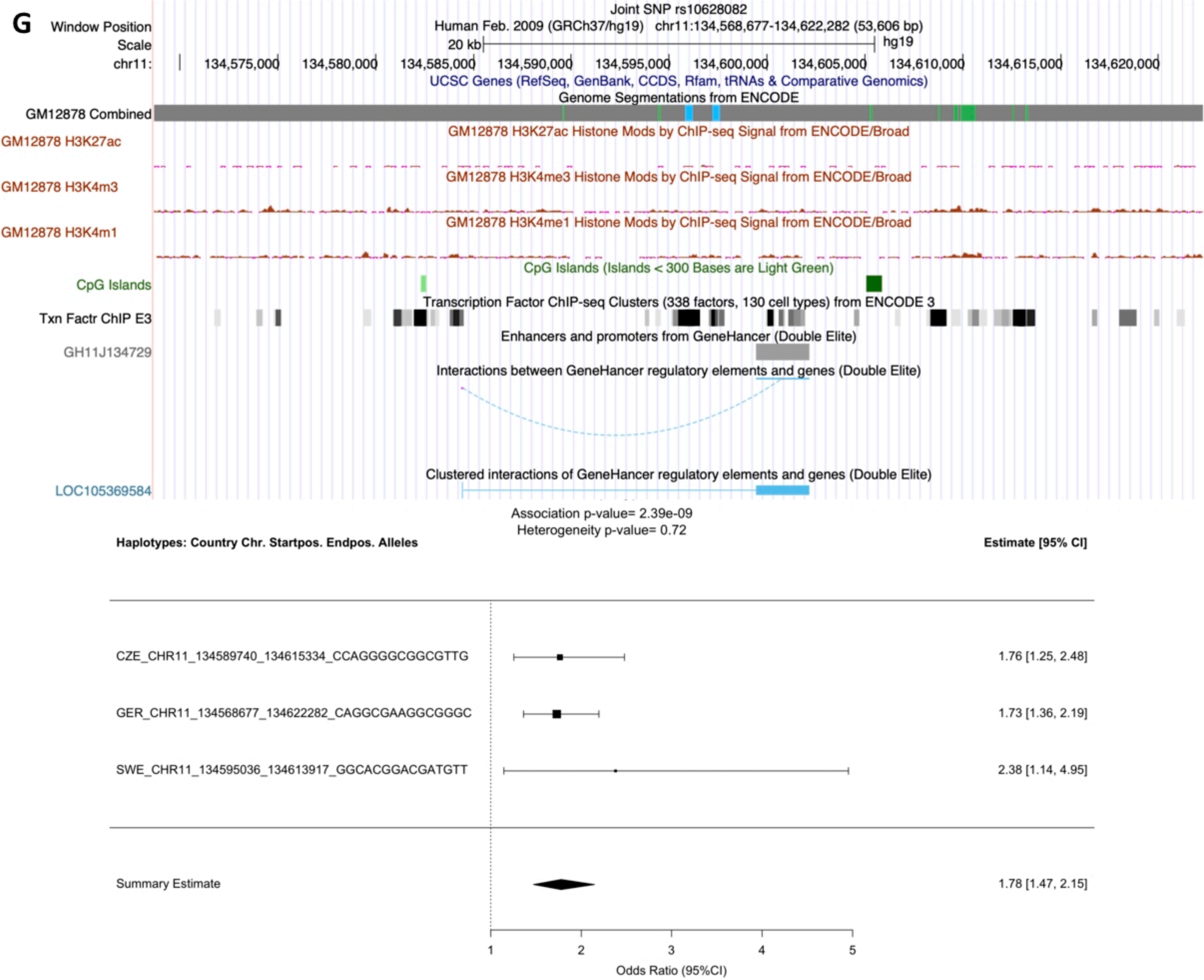
G
Joint SNP rs10628082
Window Position
Human Feb. 2009 (GRCh37/hg19) chr11:134,568,677-134,622,282 (53,606 bp)
Scale
20 kb
hg19
chr11:
134,575,000
134,580,000
134,585,000
134,590,000
134,595,000
134,600,000
134,605,000
134,610,000
134,615,000
134,620,000
UCSC Genes (RefSeq, GenBank, CCDS, Rfam, tRNAs & Comparative Genomics)
Genome Segmentations from ENCODE
GM12878 Combined
GM12878 H3K27ac Histone Mods by ChIP-seq Signal from ENCODE/Broad
GM12878 H3K27ac
GM12878 H3K4me3 Histone Mods by ChIP-seq Signal from ENCODE/Broad
GM12878 H3K4m3
GM12878 H3K4me1 Histone Mods by ChIP-seq Signal from ENCODE/Broad
GM12878 H3K4m1
CpG Islands (Islands < 300 Bases are Light Green)
CpG Islands
Transcription Factor ChIP-seq Clusters (338 factors, 130 cell types) from ENCODE 3
Txn Factr ChIP E3
Enhancers and promoters from GeneHancer (Double Elite)
GH11J134729
Interactions between GeneHancer regulatory elements and genes (Double Elite)
Clustered interactions of GeneHancer regulatory elements and genes (Double Elite)
LOC105369584
Association p-value= 2.39e-09
Heterogeneity p-value= 0.72
Haplotypes: Country Chr. Startpos. Endpos. Alleles
Estimate [95% CI]
CZE_CHR11_134589740_134615334_CCAGGGGCGGCGTTG
1.76 [1.25, 2.48]
GER_CHR11_134568677_134622282_CAGGCGAAGGCGGGC
1.73 [1.36, 2.19]
SWE_CHR11_134595036_134613917_GGCACGGACGATGTT
2.38 [1.14, 4.95]
Summary Estimate
1.78 [1.47, 2.15]
1
2
3
4
5
Odds Ratio (95%CI)

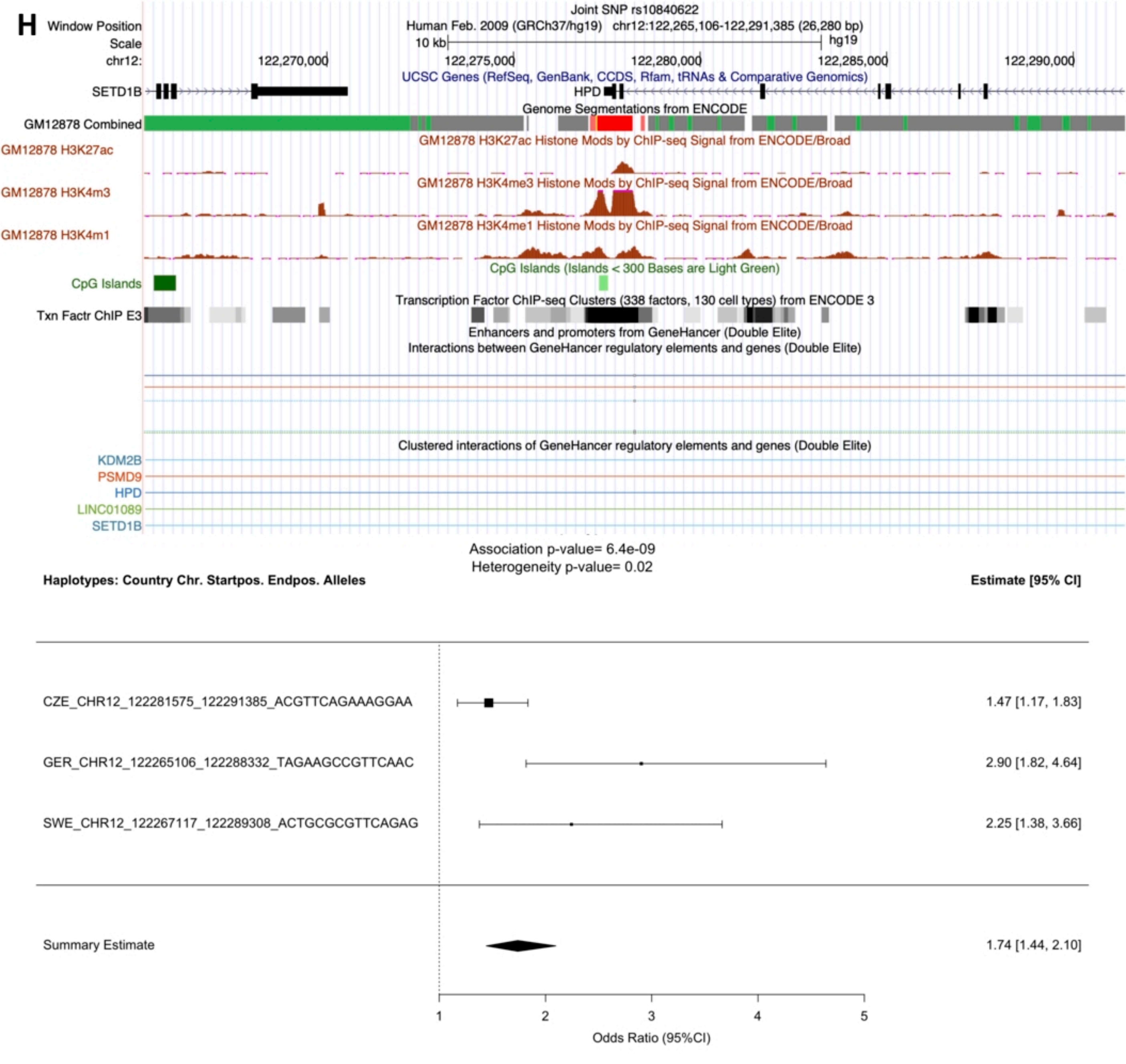
H
Joint SNP rs10840622
Window Position
Human Feb. 2009 (GRCh37/hg19) chr12:122,265,106-122,291,385 (26,280 bp)
Scale
10 kb
hg19
chr12:
122,270,000
122,275,000
122,280,000
122,285,000
122,290,000
UCSC Genes (RefSeq, GenBank, CCDS, Rfam, tRNAs & Comparative Genomics)
SETD1B
HPD
Genome Segmentations from ENCODE
GM12878 Combined
GM12878 H3K27ac Histone Mods by ChIP-seq Signal from ENCODE/Broad
GM12878 H3K27ac
GM12878 H3K4me3 Histone Mods by ChIP-seq Signal from ENCODE/Broad
GM12878 H3K4m3
GM12878 H3K4me1 Histone Mods by ChIP-seq Signal from ENCODE/Broad
GM12878 H3K4m1
CpG Islands (Islands < 300 Bases are Light Green)
CpG Islands
Transcription Factor ChIP-seq Clusters (338 factors, 130 cell types) from ENCODE 3
Txn Factr ChIP E3
Enhancers and promoters from GeneHancer (Double Elite)
Interactions between GeneHancer regulatory elements and genes (Double Elite)
Clustered interactions of GeneHancer regulatory elements and genes (Double Elite)
KDM2B
PSMD9
HPD
LINC01089
SETD1B
Association p-value= 6.4e-09
Heterogeneity p-value= 0.02
Haplotypes: Country Chr. Startpos. Endpos. Alleles
Estimate [95% CI]
CZE_CHR12_122281575_122291385_ACGTTCAGAAAGGAA
1.47 [1.17, 1.83]
GER_CHR12_122265106_122288332_TAGAAGCCGTTCAAC
2.90 [1.82, 4.64]
SWE_CHR12_122267117_122289308_ACTGCGCGTTCAGAG
2.25 [1.38, 3.66]
Summary Estimate
1.74 [1.44, 2.10]
1
2
3
4
5
Odds Ratio (95%CI)
